# Fluid Intake and Beverage Consumption Description and Their Association with Dietary Vitamins and Antioxidant Compounds in Italian Adults from the Mediterranean Healthy Eating, Aging and Lifestyles (MEAL) Study

**DOI:** 10.3390/antiox7040056

**Published:** 2018-04-09

**Authors:** Armando Platania, Dora Castiglione, Dario Sinatra, Maurizio D’ Urso, Marina Marranzano

**Affiliations:** 1 Department of Medical and Surgical Sciences and Advanced Technologies “G.F. Ingrassia”, University of Catania, 95123 Catania, Italy; armplt@hotmail.it (A.P.); doracastiglione29@gmail.com (D.C.); sinatradario@gmail.com (D.S.); 2 Provincial Health Authority of Catania, 95127 Catania, Italy; ma.durso76@gmail.com

**Keywords:** beverages, antioxidants, water, coffee, alcohol

## Abstract

The aim of the present study was to investigate the total water intake (TWI) from drinks and foods and to evaluate the correlation between the different types of drinks on energy and antioxidant intake. The cohort comprised 1602 individuals from the city of Catania in Southern Italy. A food frequency questionnaire was administered to assess dietary and water intake. The mean total water intake was 2.7 L; more than about two thirds of the sample met the European recommendations for water intake. Water and espresso coffee were the most consumed drinks. Alcohol beverages contributed about 3.0% of total energy intake, and sugar sweetened beverages contributed about 1.4%. All antioxidant vitamins were significantly correlated with TWI. However, a higher correlation was found for water from food rather than water from beverages, suggesting that major food contributors to antioxidant vitamin intake might be fruits and vegetables, rather than beverages other than water. A mild correlation was found between fruit juices and vitamin C; coffee, tea and alcohol, and niacin and polyphenols; and milk and vitamin B12. The findings from the present study show that our sample population has an adequate intake of TWI and that there is a healthy association between beverages and dietary antioxidants.

## 1. Introduction

Water comprises from 75% body weight in infants to 55% in the elderly and is an essential nutrient that is involved in all body functions—cellular homeostasis, thermoregulation and chemical reactions to name just a few. Hydration status is regulated by a complex sensitive network of physiological controls, which aims to balance water intake and loss [1]. Proper hydration status is essential in order to preserve physical and mental functions; as a matter of fact, even slight dehydration can alter these two functions [2,3]. The importance of an adequate water intake has long been recognized, and guidelines have been established by the European Food Safety Authority (EFSA). Total water intake from all sources for men and women should be about 2.5 L and 2.0 L, respectively [4]. However, it must be stated that water requirements can change according to climatic state, salt intake and physical activity as well as health status, metabolism and age. However, it has been reported that the percentage of the population with an inadequate water intake may vary from 5% to 35% among European countries [5].

Fluid intake in all age groups depends not only on water, but also on a variety of beverages, such as tea, coffee, milk, sugar-sweetened soft drinks, juice and alcoholic drinks, that may contribute, to a various extent, to the level of hydration of an individual. The percentage of water intake in a typical Western diet has been estimated to be nearly 20–30% from food and 70–80% from fluids (including water and the aforementioned beverages) [5]. Moreover, there is plenty of scientific literature regarding the investigation of the role of specific beverages on health conditions, for instance, the adverse impact of sugar-sweetened soft drinks on metabolic disorders [6,7,8], the potential beneficial effects of milk on cardiovascular diseases [9,10], the positive impact on cardio-metabolic outcomes and decreased risk of certain cancers associated with coffee [11,12] and tea consumption [13,14,15], and the increased risk of cancer related to excessive alcohol drinking [16,17]. In addition, understanding the pattern of water contribution from food and beverages as well as beverages is essential for estimating individual or population-level adherence to recommended water intakes and the appropriateness of beverage habits. In fact, it is desirable to develop specific recommendations that take into account the hydration capability and potential effects related to energy and antioxidant content of beverages [18]. A previous report [19] attempted to summarize current knowledge regarding the relationship between water intake and human health, but the authors emphasized the scarcity of scientific literature on this matter, especially with respect to how rarely fluid intake is collected in epidemiological studies. The aim of the present study was to quantify the total water and beverage intake in a cohort of Southern Italian adults in order to test their compliance with the international guidelines of the European Food Safety Authority (EFSA). Correlations between beverages, energy and dietary antioxidant intake were further explored.

## 2. Materials and Methods 

### 2.1. Study Design and Population

This study was a cross-sectional investigation of fluid intake and beverage patterns in the Mediterranean healthy eating, ageing, and lifestyle (MEAL) cohort. The total sample consisted of 2044 men and women, aged 18 to >70 years old, living in the city of Catania in Southern Italy. The sample was stratified by gender and 10-year age groups. The survey was conducted between the years 2014 and 2015; subjects and their clinical information were extracted randomly from a pool made up of general practitioners’ databases. More detailed information on the study protocol has been published elsewhere [20]. The aim of the study was explained to all participants, and a written informed consent was filled in by the subjects involved. The conduction of the study and all of its procedures were performed in accordance with the Declaration of Helsinki (1989) of the World Medical Association. The study protocol has been approved by the ethical committee of the University of Catania (protocol number: 802/23 December 2014).

### 2.2. Data Collection

The survey consisted of a face-to-face computer-assisted personal interview with tablet computers. Participants were also provided with a paper copy of the survey to read the response options, and the interviewer filled in the digital copy of the survey on the tablet computer with the final answers from the interviewee. A section of the survey collected demographic data such as gender, age at recruitment, educational status, occupation (the main source of employment during the year before the survey was collected in more detail) or last occupation before retirement, and marital status. Participants were categorized into 3 age groups: (i) 18–35 years; (ii) 35–50 years; and (iii) >50 years. Educational status was categorized in 3 groups: (i) low (primary/secondary); (ii) medium (high school); and (iii) high (university). Occupational status was categorized in 4 groups: (i) unemployed; (ii) low (unskilled workers); (iii) medium (partially skilled workers); and (iv) high (skilled workers). Physical activity status was assessed through the International Physical Activity Questionnaires (IPAQ) [21]. This is a set of questionnaires (5 domains) collecting information about the time spent practicing physical activity in the last 7 days; following the IPAQ guidelines, final scores were used to categorize physical activity level in 3 groups: (i) low; (ii) moderate; and (iii) high. Smoking status was categorized in 3 groups: (i) non-smoker; (ii) ex-smoker; and (iii) current smoker. Alcohol consumption was categorized in 3 groups: (i) none; (ii) moderate drinker (0.1–12 g/day) and (iii) regular drinker (>12 g/day). Anthropometric measurements were collected using standardized methods [22]. Height was measured to the nearest 0.5 cm without shoes, with the back square against the wall tape, eyes looking straight ahead, and a right-angle triangle resting on the scalp and against the wall. Body mass index (BMI) was calculated, and patients were categorized as under/normal weight (BMI < 25 kg/m^2^), overweight (BMI 25 to 29.9 kg/m^2^), and obese (BMI > 30 kg/m^2^) [23].

### 2.3. Dietary Assessment

Two food frequency questionnaires (a long and a short version) were administered to assess dietary and water intakes. These questionnaires have been previously tested for validity and reliability in the Sicilian population [24,25]. Beverages were combined into eight categories for further analysis: (i) alcohol beverages; (ii) sugar-sweetened beverages (including sugar-sweetened fruit juices); (iii) tea; (iv) coffee; (v) fruit juices (only 100% fruit juices); (vi) milk (all types of milk without separation by fat percentage); (vii) soy milk; (viii) water (including tap water and bottled water). Seasonal food consumption was registered during the period when the food was available; then it was adjusted by its proportional intake in one year. Food composition tables from the Research Center for Foods and Nutrition (CREA; *Consiglio per la ricerca in agricoltura e l’analisi dell’economia agraria*) [26] were used to assess the water content of food and beverages as well as energy and nutrient intakes. Total polyphenol and phytoestrogen (isoflavones and lignans) intake was calculated through a comparison with the Phenol-Explorer database (www.phenol-explorer.eu) [27]. The process of identification and calculation of the polyphenol content in foods is described in detail elsewhere [28]. Finally, intakes were adjusted for total energy intake (kcal/day) using the residual method in line with the Global Nutrition and Policy Consortium guidelines [29]. FFQs (Food Frequency Questionnaires) with unreliable intakes (<1000 or >6000 kcal/day) (*n* = 107) as well as missing items for the purposes of this study (*n* = 335) were excluded from the analyses, leaving a total of 1602 individuals included in the analysis. Total water intake (TWI) was compared with the EFSA Dietary Reference Values (DRV) regarding the Adequate Intake (AI) of water for men and women (2.5 L and 2.0 L). However, Nordic countries consider inadequate to be an intake of less than 1 g of water per 1 kcal of energy. For this reason, a combined classification was used to provide a more comprehensive evaluation of water intake: a classification based on the EFSA AI value (criterion 1), a ratio between water intake in g and energy intake in kcal (criterion 2), and a combination of criterion 1 and criterion 2 (criterion 3).

### 2.4. Statistical Analysis

Absolute numbers and percentages were used to show all frequencies, while means and standard errors were used to show continuous variables. Chi-square tests were used to compare categorical variables between groups, Student’s *t*-test and ANOVA were used to compare continuous variables distributed normally, and the Mann–Whitney *U*-test and Kruskall–Wallis test were used to compare variables that were not normally distributed. An analysis of partial correlations, adjusted for age, gender, body weight, and physical activity, was performed between water intake, energy intake, beverage consumption, vitamins and polyphenols. All analyses were based on two-sided tests and significance was set to a *p*-value of 0.05. SPSS 17 (SPSS Inc., Chicago, IL, USA) software was used for all the statistical computations.

## 3. Results

Our sample comprised 1602 participants, ranging from 18 to >70 years old, with more women than men (Table 1). One quarter were current smokers, with men being more physically active than women. On average, BMI levels suggested a certain degree of overweight individuals in the sample, though women had slightly lower BMI levels. When considering BMI in categories, there was a higher prevalence of overweight men than women, while the opposite was observed for obesity prevalence.

**Figure 1:**
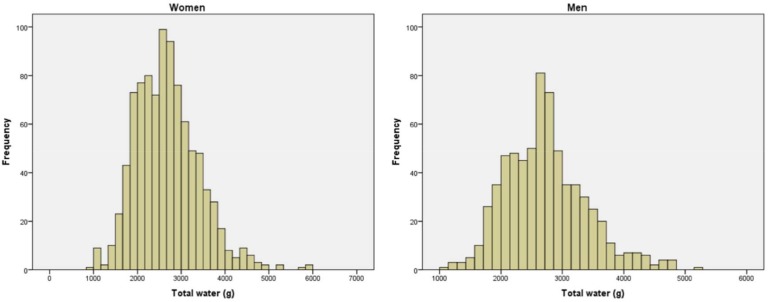
The frequency distribution of total water intake (TWI).

The frequency distribution of total water intake (TWI) showed an average of 2.7 L for both men and women (Figure 1).

The combined classification including the EFSA AI recommendations was evaluated for both sexes (Table 2). Sixty-four percent of men and 83% of women met EFSA criterion 1 (total water intake >2.5 L in men, >2.0 L in women), while 88% of men and 86% of women met EFSA criterion 2 (water/energy intake >1).

**Figure 2:**
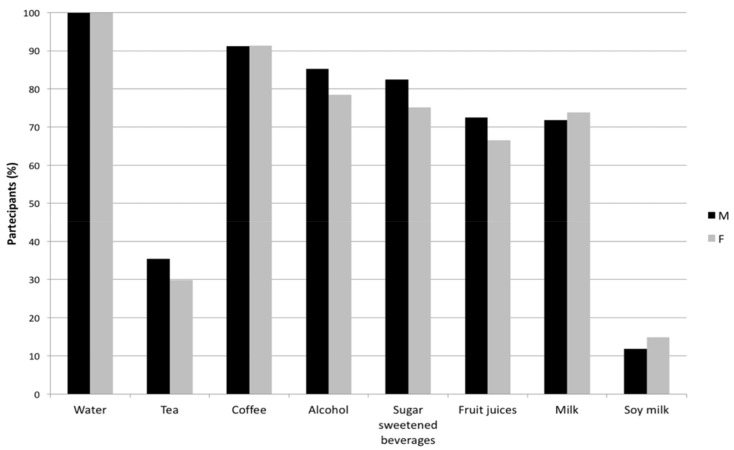
Prevalence of beverage consumption (% consumption by males and females).

The major contribution to TWI was given by total beverages (73%), for which preference of consumption was assessed in both men and women (Figure 2). Water was consumed by all participants, followed by coffee (91% by both genders), alcohol beverages (85% by men, 79% by women) and sugar-sweetened beverages (83% by men, 75% by women). Tea and soy milk beverages were the less preferred, being consumed, respectively, by 35% of men and 30% of women (tea), and 12% of men and 15% of women (soy milk).

Percentages of total weight (food and beverages) consumed (g/day), daily energy intake (kcal/day), and water intake (g/day) were calculated (Table 3). Regarding TWI, 73% came from beverages and 27% from food. These percentages met the EFSA recommendations regarding the percentage of water intake that should be provided by beverages (70–80%) and food (20–30%). Furthermore, water was the main source of TWI with a score of 55%. The mean total energy intake was 1951 kcal/day, with a relative contribution of 9% from beverages. Of these, alcohol beverages contributed 3.0% for men and 2.7% for women, while sugar-sweetened beverages contributed 1.4% for men and 1.3% for women. Little difference was found between the total weight percentage of alcohol beverages consumed by men and women (respectively, 3.5% and 3.0%).

Water intake was also calculated separately by age group (Table 4). 

The amount of water consumed in beverages did not change with age, while alcohol beverage consumption was higher in the elderly, followed by younger participants and then middle-aged ones. TWI correlated highly with the consumption of water from beverages (*r* = 0.82) and mildly with food weight (*r* = 0.68) (Table 5).

Alcohol beverages, milk and sugar-sweetened beverages had the highest correlations with total energy from beverages (*r* = 0.64, *r* = 0.51, and *r* = 0.47, respectively), while the scores were low for the other beverages and near zero for water and tea. All antioxidant vitamins were significantly correlated with TWI (Table 6).

However, a higher correlation with TWI was found for water from food than for water from beverages, suggesting that the major food contributors to antioxidant vitamin intake might be fruits and vegetables, rather than beverages other than water. Another strong correlation was found between total phytoestrogen intake and soy milk intake (*r* = 0.78), suggesting that soy milk is a major contributor to phytoestrogen intake in this cohort. A mild correlation was found between fruit juices and vitamin C (*r* = 0.42), and tea and polyphenols (*r* = 0.37), and other correlations were found between coffee, and niacin (*r* = 0.31) and polyphenols (*r* = 0.20); milk and vitamin B12 (*r* = 0.32); and alcohol and polyphenols (*r* = 0.31).

## 4. Discussion

This study analysed the total water intake from both food and beverages among the MEAL cohort, a representative sample of Southern Italian adults. Our study is one of the few attempts to describe the consumption of water and beverages in Italy, the relationship between energy intake and beverages, as well as being a reference for the role of some antioxidant vitamins and polyphenols contained in beverages. The EFSA Scientific Opinion on Dietary Reference Values for Water states that the dietary reference intake values for water should include water from drinking water (tap or bottled), all kinds of beverages, and food moisture. The adequate total water intake suggested by EFSA guidelines (2 L for women and 2.5 L for men) was met by the majority of our sample population.

Comparisons with similar studies are not easy to perform due to the use of different populations with different dietary habits, as well as different tools administered. Nonetheless, it can be stated that participants from two previous studies on hydration (Spanish and Italian) did not reach the EFSA guidelines recommended intake for water, while participants in another one (English) did reach the recommended intake [30,31,32]. In our study, the main contributor to TWI was plain water. Our results are only partially in line with previous studies in which the main source of TWI was either food (Italy and Spain) or hot beverages (UK); this can be explained by the hotter seasonal temperatures in Southern Italy, which stimulate thirst induction, and the preference for plain water over sugar-sweetened beverages due to the cultural heritage of adults living in Southern Italy who are still adherent, at least in part, to traditional dietary patterns [33,34]. The main benefit from a hydration pattern which mainly focuses on plain water intake is the fulfillment of its purpose without adding caloric intake, while providing mineral micronutrients. In line with other studies [30,31,32], water intake from beverages decreased with age, underlining a common trend which sees the elderly drinking less. Evidence from studies conducted in Mediterranean populations shows that adherence to traditional dietary patterns (including beverage consumption) might be beneficial for body composition in young, adult and elderly individuals [35,36,37]. However, more studies are needed to evaluate whether this trend could be linked with a risk of dehydration in the elderly or if it is a harmless manifestation of the lower physiological water needs of the metabolism in late years.

The second most popular beverage was coffee, namely as espresso coffee, which was drunk by 91% of our sample, but still had a low contribution to TWI (2%) due to the small quantity contained in a serving. In our analysis, we found a statistically significant correlation between coffee and niacin and total polyphenols. Coffee contains around 5 mg of niacin and thus can be an important source of this vitamin [38]. Moreover, previous studies have demonstrated that coffee is among the highest source of polyphenols in individuals living in Northern and Eastern European populations [39,40] due to the large intake compared with individuals living in Southern European countries, such as France, Spain and Italy [41,42,43]. Coffee drinking has been associated with positive health outcomes [44]. Regarding espresso coffee, despite it being reported to provide an important antioxidant capacity within the context of a common diet [45,46], results regarding its effects on health are varied, ranging from positive [47,48,49] to negative [50,51,52]. In general, scientific literature on espresso coffee is scarce, and further research is needed to test the impact of this beverage on human health.

Regarding alcoholic beverage consumption, women almost reached the same intake as men (3.0% vs. 3.5%) in contrast with previous studies in which men had, in some cases, double the consumption of women [32]. Regular and moderate daily alcohol consumption is a typical pattern of the Mediterranean diet, with wine being the most consumed type of alcoholic beverage [53]. This habit is especially rooted in the elderly, as confirmed by our analysis stratified by age groups. Furthermore, a small correlation has been found between alcohol beverages and polyphenols. The caloric contribution of alcohol beverages was 3%; the downside of the non-negligible caloric intake of alcoholic beverages could be softened by the benefits of moderate alcohol use. In fact, there is evidence that dietary polyphenols may exert beneficial effect toward human health [54,55]. It has been established that alcohol has a protective effect at low doses, in particular, for red wine in regard to cardiovascular diseases, and the presence of polyphenols contained in such beverages has been proposed as one of the main contributors to this effect [56,57]. Another source of polyphenols is tea, as remarked by our correlation analysis. However, its consumption was relatively low in our sample, comprising 2% of total water weight consumed, with a popularity of only 30% of the population. Finally, the least popular beverage was soy milk, which was highly correlated with phytoestrogen intake. Soy foods and beverages are hardly consumed in the Mediterranean area compared to Asian countries [58], but evidence suggests that phytoestrogens, such as isoflavones, may exert beneficial effects on health [59]. A previous study involving the present cohort showed certain positive associations between phytoestrogen consumption and hypertension despite the low consumption [34], suggesting that this beverage has the potential for dietary recommendation in Mediterranean countries and could be taken into account for future dietary strategies. Besides polyphenols, antioxidant vitamins were correlated with total water intake, and specifically with water from food. This suggests that foods with a high content of water, like fruits and some kinds of vegetables, are the main source of these substances [60,61,62]. As expected, correlations between vitamin C and fruit juices, and vitamin B12 and milk were found, suggesting that such beverages are among the major sources of these vitamins.

Beverages’ contribution to total energy intake was 9%, less than the 10% suggested by EFSA and WHO (World Health Organization) [4,63]. It has been recently discussed worldwide whether energy intake from beverages could have a role in metabolic disorders such as obesity [64,65,66,67]. According to recent scientific literature, there is a wide difference in beverage energy contribution between countries, ranging from around 5–10% in Italy and Spain, to 16% in the UK and 21% in the US [30,31,32,68]. The habit of drinking sugar-sweetened beverages is not common in the context of a Mediterranean diet pattern, as reported in our results (sugar-sweetened beverages accounted for only 1.3% of total caloric intake), as well as findings from other Italian and Spanish studies (0.4–2%) [30,31]. There is evidence from this cohort that traditional dietary patterns may be associated with a lower likelihood of having metabolic disorders [69,70,71]. In contrast, studies conducted in the UK and the US reported alarming trends in sugar-sweetened beverage consumption, which have been ascribed as being potentially responsible for the rise in metabolic disorders in these areas [32,68].

The main limitation of this study was the lack of a hydration biomarker that could definitely correlate fluid intake with the hydration levels of the individuals involved in the survey. Second, the cross-sectional nature of the study design and the use of a FFQ to estimate dietary information may lead to recall bias, with some overestimations (for instance on water intakes) and underestimations (alcohol beverages).

## 5. Conclusions

In conclusion, the results of the present study show that our sample population had adequate intake of TWI in accordance with EFSA reference values. Caloric beverages did not have a big impact on total caloric intake among the sample population, entering the boundaries of EFSA and WHO recommendation limits. Some beverages, like fruit juices, can cause a significant intake of certain vitamins and could be included in specific recommendations to avoid nutritional deficiencies. A small number of studies have investigated hydration status on a population level, thus further investigations are needed to identify trends and issues that may require specific health and nutrition policies or targeted interventions.

## Figures and Tables

**Table 1:** Descriptive statistics of the Mediterranean healthy eating, ageing, and lifestyle (MEAL) sample.

	Male (*n* = 669) (41.8% column)	Female (*n* = 933) (58.2% column)	Total (*n* = 1602)
Age groups, *n* (%)			
18–35	210 (31.4)	260 (27.9)	470 (29.3)
35–50	199 (29.7)	273 (29.3)	472 (29.5)
>50	260 (38.9)	400 (42.9)	660 (41.2)
Education, *n* (%)			
Elementary or Middle school	157 (23.5)	314 (33.7)	471 (29.4)
Diploma	308 (46.0)	341 (36.5)	649 (40.5)
Graduate	204 (30.5)	278 (29.8)	482 (30.1)
Occupation, *n* (%)			
Unemployed	66 (11.7)	299 (37.8)	365 (26.9)
Low (unskilled workers)	96 (17.1)	122 (15.4)	218 (16.1)
Medium (partially skilled workers)	170 (30.2)	153 (19.3)	323 (23.8)
High (skilled workers)	231 (41.0)	218 (27.5)	449 (33.1)
Smoking, *n* (%)			
Yes	173 (25.9)	225 (24.1)	398 (24.8)
No	374 (55.9)	648 (69.5)	1022 (63.8)
Ex-smoker	122 (18.2)	60 (6.4)	182 (11.4)
Body Mass Index (BMI) (kg/m^2^) mean (SE)	26.3 (3.7)	25.1 (4.9)	25.6 (4.5)
BMI category (kg/m^2^) *n* (%)			
Underweight/Normal (<18.5–24.9)	237 (39.4)	486 (55.7)	723 (49.1)
Overweight (25–29.9)	280 (46.5)	231 (26.5)	511 (34.7)
Obese (>29.9)	85 (14.1)	155 (17.8)	240 (15.0)
Physical activity (scale) *n* (%)			
Low	76 (11.4)	201 (21.5)	277 (17.3)
Moderate	317 (47.4)	481 (51.6)	798 (49.8)
High	276 (41.3)	247 (26.5)	523 (32.6)
Total Water Intake (L) mean (SE)	2729 (25.4)	2682 (23.6)	2701 (17.4)

**Table 2:** Combined classification for total water intake (TWI) following established criteria.

Criteria Classification	Men (*n* = 669)	Women (*n* = 933)
Criterion 1: (%)	63.7	82.7
Criterion 2: (%)	88.3	86.4
Criterion 3: (%)	59.8	75.1

EFSA: European Food Safety Authority. (1) Criterion 1: TWI >2.5 L men, >2 L women; (2) Criterion 2: ratio of total water intake and total energy >1; (3) Criterion 3: both criteria 1 and 2.

**Table 3:** Contribution of food and beverages to total water and energy intake.

	Total Weight Consumed (g/day)			Contribution to Energy Intake (kcal/day)			Contribution to Water Intake (g/day)		
	Total	Men	Women	Total	Men	Women	Total	Men	Women
All food and drink, mean (SE)	3072.70	3102.36	3051.43	1950.53	1975.22	1932.83	2701.71	2729.08	2682.08
	(19.76)	(28.07)	(27.31)	(16.89)	(24.81)	(22.90)	(17.35)	(25.40)	(23.57)
Food only, mean (SE)	36.0%	35.3%	36.5%	91.3%	91.4%	91.2%	27.4%	26.6%	28.0%
	(0.3%)	(0.4%)	(0.3%)	(0.1%)	(0.2%)	(0.2%)	(0.3%)	(0.4%)	(0.3%)
Beverages only, mean (SE)	64.0%	64.7%	63.5%	8.7%	8.6%	8.8%	72.6%	73.4%	72.0%
	(0.3%)	(0.4%)	(0.3%)	(0.1%)	(0.2%)	(0.2%)	(0.3%)	(0.4%)	(0.4%)
Alcohol beverages, mean (SE)	3.2%	3.5%	3.0%	2.8%	3.0%	2.7%	3.7%	4.0%	3.5%
	(0.1%)	(0.2%)	(0.2%)	(0.1%)	(0.1%)	(0.1%)	(0.1%)	(0.2%)	(0.2%)
Sugar-sweetened beverages, mean (SE)	2.1%	2.2%	2.0%	1.3%	1.4%	1.3%	2.5%	2.6%	2.4%
	(0.1%)	(0.1%)	(0.1%)	(0.1%)	(0.1%)	(0.1%)	(0.1%)	(0.2%)	(0.2%)
Tea, mean (SE)	2.4%	2.0%	2.7%	0.0%	0.0%	0.0%	2.7%	2.3%	3.0%
	(0.1%)	(0.2%)	(0.1%)	(0.0%)	(0.0%)	(0.0%)	(0.1%)	(0.2%)	(0.2%)
Coffee, mean (SE)	1.9%	1.9%	1.9%	0.3%	0.3%	0.3%	2.1%	2.2%	2.1%
	(0.0%)	(0.1%)	(0.0%)	(0.0%)	(0.0%)	(0.0%)	(0.0%)	(0.1%)	(0.1%)
Fruit juices, mean (SE)	1.1%	1.2%	1.1%	0.7%	0.8%	0.7%	1.3%	1.4%	1.2%
	(0.0%)	(0.1%)	(0.1%)	(0.0%)	(0.1%)	(0.0%)	(0.1%)	(0.1%)	(0.1%)
Milk, mean (SE)	4.3%	4.0%	4.5%	3.2%	2.9%	3.4%	4.9%	4.5%	5.2%
	(0.1%)	(0.2%)	(0.2%)	(0.1%)	(0.1%)	(0.1%)	(0.1%)	(0.2%)	(0.2%)
Soy milk, mean (SE)	0.5%	0.3%	0.6%	0.3%	0.2%	0.4%	0.5%	0.3%	0.7%
	(0.0%)	(0.0%)	(0.1%)	(0.0%)	(0.0%)	(0.0%)	(0.0%)	(0.0%)	(0.1%)
Water, mean (SE)	48.4%	49.5%	47.7%	/	/	/	54.9%	56.2%	54.0%
	(0.3%)	(0.4%)	(0.4%)	/	/	/	(0.3%)	(0.5%)	(0.4%)

**Table 4:** Total water intake and beverage consumption (g/day) by age group.

	Men				Women			
	Age Group			*p*	Age Group			*p*
	1	2	3		1	2	3	
**Total water intake from food and beverages**, mean (SE)	2804	2676	2709	0.12	2686	2739	2640	0.22
	53	43	37		43	44	36	
**Water from food**, mean (SE)	745	720	738	0.77	770	772	755	0.83
	30	24	19		23	30	18	
**Water from beverages**, mean (SE)	2059	1956	1971	0.07	1916	1967	1885	0.11
	40	32	29		32	32	26	
**Alcoholic beverages**, mean (SE)	112	91	127	0.05	93	78	98	0.20
	9	7	12		8	7	8	
**Sugar-sweetened beverages**, mean (SE)	84	65	66	0.18	76	71	55	0.13
	9	8	7		9	8	6	
**Tea**, mean (SE)	84	60	67	0.34	74	102	84	0.10
	13	11	10		7	12	7	
**Coffee**, mean (SE)	52	57	61	0.07	53	50	56	0.14
	3	3	3		3	2	2	
**Fruit juices**, mean (SE)	39	32	44	0.10	36	41	31	0.19
	4	3	5		4	5	3	
**Milk**, mean (SE)	126	117	114	0.68	129	144	132	0.52
	10	11	9		10	11	8	
**Soy milk**, mean (SE)	11	9	7	0.48	27	25	16	0.10
	3	3	2		5	5	3	
**Water**, mean (SE)	1552	1525	1485	0.22	1428	1456	1413	0.48
	31	29	26		27	27	24	

*p*-values were obtained through ANOVA tests.

**Table 5:** Partial correlations between water intake, energy intake and beverage consumption.

	Total Water	Water from Beverages	Water from Food	Food Weight	Total Energy	Energy from Beverages	Energy from Food
Total water	1.00	0.82 **	0.66 **	0.68 **	0.53 **	0.37 **	0.49 **
Water from beverages	0.82 **	1.00	0.12 **	0.18 **	0.25 **	0.40 **	0.19 **
Water from food	0.66 **	0.12 **	1.00	0.96 **	0.60 **	0.11 **	0.61 **
Food weight	0.68 **	0.18 **	0.96 **	1.00	0.73 **	0.22 **	0.73 **
Total energy	0.53 **	0.25 **	0.60 **	0.73 **	1.00	0.39 **	0.98 **
Energy from beverages	0.37 **	0.40 **	0.11 **	0.22 **	0.39 **	1.00	0.22 **
Energy from food	0.49 **	0.19 **	0.61 **	0.73 **	0.98 **	0.22 **	1.00
Alcohol beverages	0.22 **	0.23 **	0.08 **	0.10 **	0.21 **	0.64 **	0.10 **
Sugar-sweetened beverages	0.20 **	0.24 **	0.05 *	0.20 **	0.35 **	0.47 **	0.28 **
Tea	0.34 **	0.23 **	0.16 **	0.15 **	0.03	0.06 *	0.02
Coffee	0.08 **	0.06 *	0.05 *	0.03	0.07 **	0.14 **	0.05 *
Fruit juices	0.29 **	0.21 **	0.22 **	0.27 **	0.18 **	0.28 **	0.14 **
Milk	0.12 **	0.20 **	−0.04	0.00	0.12 **	0.51 **	0.03
Soy milk	0.16 **	0.14 **	0.09 **	0.10 **	0.05 *	0.10 **	0.04
Water	0.61 **	0.81 **	0.02	0.02	0.03	−0.14 **	0.06 *

** Correlation is significant at the 0.01 level (bilateral); * Correlation is significant at the 0.05 level (bilateral).

**Table 6:** Partial correlations between water intake, energy intake, beverage consumption, vitamins and polyphenols.

Title	Total Water	Water from Beverages	Water from Food	Food Weight	Total Energy	Energy from Beverages	Energy from Food	Alcohol Beverages	Sugar-Sweetened Beverages	Tea	Coffee	Fruit Juices	Milk	Soy Milk	Water
Vitamin A	0.60 **	0.21 **	0.75 **	0.74 **	0.54 **	0.25 **	0.52 **	0.11 **	0.01	0.23 **	0.05	0.21 **	0.01	0.15 **	0.03
Vitamin B1	0.48 **	0.22 **	0.54 **	0.56 **	0.76 **	0.27 **	0.75 **	0.15 **	0.23 **	0.15 **	0.05	0.09 **	0.08 **	0.11 **	0.07 **
Vitamin B2	0.51 **	0.27 **	0.52 **	0.53 **	0.72 **	0.39 **	0.69 **	0.17 **	0.19 **	0.18 **	0.12 **	0.06 *	0.27 **	0.17 **	0.04
Niacin	0.54 **	0.27 **	0.60 **	0.69 **	0.77 **	0.25 **	0.76 **	0.12 **	0.16 **	0.11 **	0.31 **	0.10 **	0.00	0.14 **	0.10 **
Vitamin B6	0.66 **	0.29 **	0.73 **	0.82 **	0.83 **	0.30 **	0.82 **	0.08 **	0.12 **	0.25 **	0.02	0.17 **	0.05 *	0.18 **	0.08 **
Folate	0.59 **	0.23 **	0.73 **	0.79 **	0.69 **	0.25 **	0.68 **	0.12 **	0.06 *	0.20 **	0.06 *	0.23 **	0.01	0.21 **	0.06 *
Vitamin B12	0.35 **	0.21 **	0.35 **	0.41 **	0.44 **	0.14 **	0.44 **	0.06 *	0.27 **	0.11 **	−0.03	0.11 **	0.32 **	0.04	0.20 **
Vitamin C	0.62 **	0.18 **	0.84 **	0.85 **	0.50 **	0.29 **	0.47 **	0.12 **	0.06 *	0.18 **	0.02	0.42 **	0.00	0.13 **	−0.03
Vitamin D	0.31 **	0.17 **	0.32 **	0.36 **	0.35 **	0.04	0.37 **	−0.02	0.15 **	0.14 **	−0.05	0.14 **	0.06*	0.10 **	0.20 **
Vitamin E	0.57 **	0.21 **	0.71 **	0.74 **	0.76 **	0.28 **	0.76 **	0.15 **	0.14 **	0.19 **	0.01	0.15 **	−0.05 *	0.19 **	0.02
Total polyphenols	0.46 **	0.24 **	0.43 **	0.44 **	0.42 **	0.32 **	0.39 **	0.31 **	−0.07 **	0.37 **	0.20 **	0.13 **	−0.11 **	0.14 **	0.04
Total phytoestrogens	0.34 **	0.14 **	0.40 **	0.39 **	0.38 **	0.05 *	−0.05 *	0.15	0.01	0.12 *	−0.05 *	0.11 **	-0.08 **	0.78 **	0.04

** Correlation is significant at the 0.01 level (bilateral); * Correlation is significant at the 0.05 level (bilateral).
